# PAR6, A Potential Marker for the Germ Cells Selected to Form Primordial Follicles in Mouse Ovary

**DOI:** 10.1371/journal.pone.0007372

**Published:** 2009-10-07

**Authors:** Jing Wen, Hua Zhang, Ge Li, Guanping Mao, Xiufen Chen, Jianwei Wang, Meng Guo, Xinyi Mu, Hong Ouyang, Meijia Zhang, Guoliang Xia

**Affiliations:** State Key Laboratory for Agrobiotechnology, College of Biological Sciences, Agricultural University, Beijing, People's Republic of China; The University of Hong Kong, China

## Abstract

Partitioning-defective proteins (PAR) are detected to express mainly in the cytoplast, and play an important role in cell polarity. However, we showed here that PAR6, one kind of PAR protein, was localized in the nuclei of mouse oocytes that formed primordial follicles during the perinatal period, suggesting a new role of PAR protein. It is the first time we found that, in mouse fetal ovaries, PAR6 appeared in somatic cell cytoplasm and fell weak when somatic cells invaded germ cell cysts at 17.5 days post coitus (dpc). Meanwhile, the expression of PAR6 was observed in cysts, and became strong in the nuclei of some germ cells at 19.5 dpc and all primordial follicular oocytes at 3 day post parturition (dpp), and then obviously declined when the primordial follicles entered the folliculogenic growth phase. During the primordial follicle pool foundation, the number of PAR6 positive germ cells remained steady and was consistent with that of formed follicles at 3 dpp. There were no TUNEL (apoptosis examination) positive germ cells stained with PAR6 at any time studied. The number of follicles significantly declined when 15.5 dpc ovaries were treated with the anti-PAR6 antibody and PAR6 RNA interference. Carbenoxolone (CBX, a known blocker of gap junctions) inhibited the expression of PAR6 in germ cells and the formation of follicles. Our results suggest that PAR6 could be used as a potential marker of germ cells for the primordial follicle formation, and the expression of PAR6 by a gap junction-dependent process may contribute to the formation of primordial follicles and the maintenance of oocytes at the diplotene stage.

## Introduction

In mice, the establishment of the primordial follicle pool is a complex process that includes the formation of cysts through oogonia mitosis, the startup of initial meiosis, the breakdown of cysts, and the formation of primordial follicles when germ cells are arrested at the diplotene stage [1]. During this process, germ cells in fetal ovaries can develop to form primordial follicles or undergo apoptosis, which depends on molecular regulatory mechanism that remains elusive, for example, why can only a few oocytes cooperate with somatic cells to form primordial follicles, which kind of germ cells are selected to form the primordial follicles with ovarian somatic cells, why can not the oocytes finish their first meiosis and arrest at the diplotene stage, which factors control these?

PAR proteins play an important role in cell polarity of cells of many types. They are involved in the asymmetric distribution of cytoplasmic determinants and in the regulation of cytoskeleton positioning and asymmetric division. The core in PAR protein is a ternary complex of atypical protein kinase C (aPKC), the PDZ-domain proteins PAR-3 and PAR-6. Two others are protein kinases called Par-1, and Par-5, which belong to the 14-3-3 family of phosphoserine-binding proteins [2]. Their localizations are mutually exclusive which may provide a general mechanism to establish cortical domains in polar cells.

In *Drosophila*, data show that the polarisation of the germ cell can be traced back to much earlier stages of oogenesis. Cystoblasts divide 4 times followed by incomplete cytokinesis to form a germline cyst of 16 connected cells, comprising a single oocyte and 15 nurse cells, which provide the oocyte with nutrients and cytoplasmic components [3], [4]. As to form primordial follicle, only the germ cell which express the par protein can become the oocyte and others undergo degeneration. Any mutant of the conserved Par proteins would disrupt the early polarisation of the oocyte and lead to failure to maintain its identity [5]–[10]. Furthermore, data elucidate that the mutations in the par genes and cell cycle regulators such as dacapo [11] could produce strikingly similar phenotypes.

In mice, the expression of PAR proteins was observed in oocytes during meiotic maturation (PAR3, PAR6) [12], [13], and in the 8-cell stage of preimplantation embryo during compaction (PAR1, PAR6) [14]. However, PAR proteins in the early stages of mouse oogenesis have not been reported. The objective of this study was to investigate the expressive character of PAR6 and its role during primordial follicle formation in mice.

## Results and Discussion

### PAR6 expressive character during primordial follicle formation in mice

**Figure 1:**
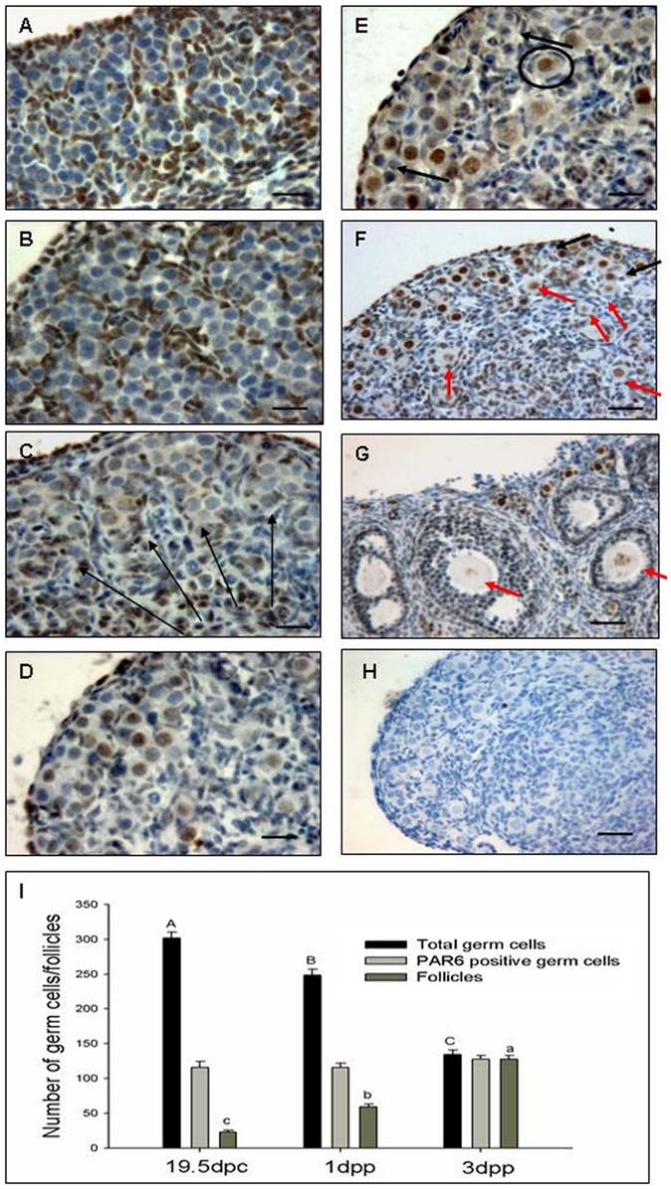
Immunohistochemical localization of PAR6 in the mouse ovary. The germ cells with the cyst structure in the 13.5 dpc (A) and 15.5 dpc (B) fetal ovaries did not show positive staining for PAR6 in contrast to the strong staining in somatic cells. At 17.5 dpc, the germ cells moved to the cortex (long arrow), and their cytoplasm began to express PAR6 (C). After 19.5 dpc, PAR6 staining was strong in the nuclei of some germ cells (D, 19.5 dpc; E, 1 dpp) and almost all oocytes in primordial follicles, while the oocytes nuclei of growing follicles showed weak expression of PAR6 (F, 3 dpp; G, 8 weeks). No reaction was observed in control sections (3 dpp) after replacement of the anti-PAR6 antibody with nonimmune serum (H). Black arrow, small or shrunk germ cell; red arrow, growing follicle; ring, primordial follicle. A–E, Bar = 20 µm; F–H, Bar = 40 µm. The numbers of germ cells, PAR6 positive germ cells and follicles in the largest cross-section of the ovary with different stages (I). Each independent data came from six ovaries. Different superscripts denote differences (P<0.05) in the total germ cells or follicles, respectively.

By immunohistochemistry, we examined the expression of PAR6 during the early stages of mouse ovary development. The results demonstrated for the first time that PAR6 was expressed in the mouse fetal ovary with a spatio-temporal character (Fig. 1). In most mouse strains, primordial germ cells arrive at the genital ridge at 10 dpc, embark on a special program of division to form cysts at 10.5 dpc, start meiosis at 13.5 dpc through the stages of leptotene and zygotene, and then enter the pachytene stage at 15.5 dpc [15]. The expression of PAR6 was high in the cytoplasm of somatic cells but was not observed in germ cells during these stages (Fig. 1A,B, and data not shown). From 17.5 dpc, the germ cells enter the diplotene stage from the pachytene stage [15], and the somatic cells begin to incorporate cysts in order to form primordial follicles at an appropriate time [16], [17]. At the same time, the germ cells migrated to the cortex and expressed PAR6 lowly within the cytoplasm of cysts, whereas the expression in somatic cells around the cysts turned weak (Fig. 1C). The expression of this polarity protein in the cysts was likely to be relevant to the oocyte recognition and incorporation by the somatic cells as that in Drosophila [18].

The reduction of the germ cell number and the formation of the primordial follicles are the key occurrences in the perinatal ovaries, At 19.5 dpc, most of germ cells were still in cysts and PAR6 began to accumulate in the nuclei of germ cells. Moreover, in every cyst, there were only certain germ cells which can express PAR6 (Fig. 1D). We have stained the adjacent section with MVH (a germ cell marker) to make sure that the germ cells we defined were really germ cells (Fig. S1). At 1 dpp, primordial follicles were observed on the basis of their association with a surrounding layer of squamous-type granulosa cells (Fig. 1E, ring), in which the nuclei of oocytes showed intense PAR6 staining, whereas the smaller germ cells showed weak or no labeling (Fig. 1E, black arrow).

At 3 dpp, the primordial follicular pool is established gradually [19]. In our study, almost all of the germ cells in the cortex,in which the nuclei of oocytes expressed PAR6 highly could be classified as primordial follicles. Only a few of germ cells had not formed primordial follicles, which showed negative PAR6 staining and degenerate characteristics: small size and shrink (Fig. 1F, black arrow, and data not shown). The primary follicles surrounded by cubical-type granulosa cells were observed in the medulla (Fig. 1F), and the expression of PAR6 became weak in the oocyte nuclei (Fig. 1F, red arrow). These phenomena were also observed in the ovaries of mature mice, (Fig. 1G, 8 weeks old). However, the expression of PAR6 was not detected in all stages of prespermatogonial and spermatogonial developments (Fig. S2).

During the establishment of the primordial follicular pool, the number of germ cells decreased sharply [16], [20], since a large number of germ cells that could not form primordial follicles had undergone apoptosis. We counted the numbers of total germ cells, PAR6 positive germ cells and follicles in the section from the largest cross-section through the center of the ovary at 19.5 dpc, 1 dpp and 3 dpp. The number of germ cells declined (19.5 dpc, 302±9; 1 dpp, 248±8; 3 dpp, 136±6. P<0.05, respectively. Fig. 1I), the number of follicles increased (19.5 dpc, 23±3; 1 dpp, 59±4; 3 dpp, 129±5. P<0.05, respectively. Fig. 1I), but the PAR6 positive germ cells remained steady (19.5 dpc, 116±8; 1 dpp, 116±6; 3 dpp, 129±5. Fig. 1I). At 3 dpp, the number of follicles was the same as that of PAR6 positive germ cells since the germ cells in follicles showed positive staining, and others without follicular structures showed negative staining. Primordial follicle forming starts from the 17.5 dpc [21], and about one third of germ cells contribute to the establishment of the primordial folliclular pool by 3 or 4 dpp [17], [19], which endow all the available follicles for reproduction of animals. The peak of germ cell apoptosis occurs at prenatal, as well as neonatal when germ cells transit to the meiotic diplotene stage from the pachytene stage [17], [20]. In our study, about one third, half and almost all of germ cells expressed PAR6 at 19.5 dpc, 1 dpp and 3 dpp, respectively (Fig. 1I), but the number of PAR6 positive germ cells was constant during these stages. Several germ cells without PAR6 expression at 3 dpp had not formed primordial follicles and presented the degenerate characteristics. All these results indicate that the germ cells with PAR6 expression may have the ability to form primordial follicles in mice.

The stage of PAR first presence in oogenesis is a crucial developmental checkpoint in Drosophila oocytes, and the mutations in the par genes or cell cycle regulators produce similar phenotypes [11], demonstrating that PAR proteins may have a correlation with the regulation of cell cycle. In mice, the reduction in germ cell number during the primordial follicle formation is a regulated developmental process [17]. The strong expression of PAR6 in some germ cells during this brief period may be involved in this regulated process and associated with cell cycle regulators to make sure that the germ cells arrested at the diplotene stage can form primordial follicles. In this point, PAR6 may serve as a potential marker for the germ cell selection.

It is reported that PAR protein members are organized as homo- or hetero- complexes which localize to specific domains in the cytoplasm. For instance, PAR1 localizes to the posterior cortex of the one-cell Caenorhabditis elegans embryo and the Drosophila oocyte [22], [23], while PAR6, aPKC and PAR3 complexes localize either to the anterior cell cortex in Caenorhabditis elegans and Drosophila or to adherence junctions in Drosophila epithelia and tight junctions in mammalian epithelia [24]. In our study, the PAR6 was strongly expressed in the nuclei of oocytes when the germ cells entered and were arrested at the diplotene stage to form primordial follicles, and kept for a long time until the primordial follicles were recruited into the population of growing follicles. We speculated that the PAR6 located in the nuclei of oocytes is involved in organizing the higher order chromatin structures by crosslinking chromatin subunits during the cell cycle of meiosis suspended state [25] and maintaining the stability of the primordial follicular pool. The possible reason for negative staining in male germ cells is that there is no incorporation of the somatic cells and the meiosis arrested phase in sperm genesis.

### The germ cells with PAR6 expression did not show apoptosis characteristics

**Figure 2:**
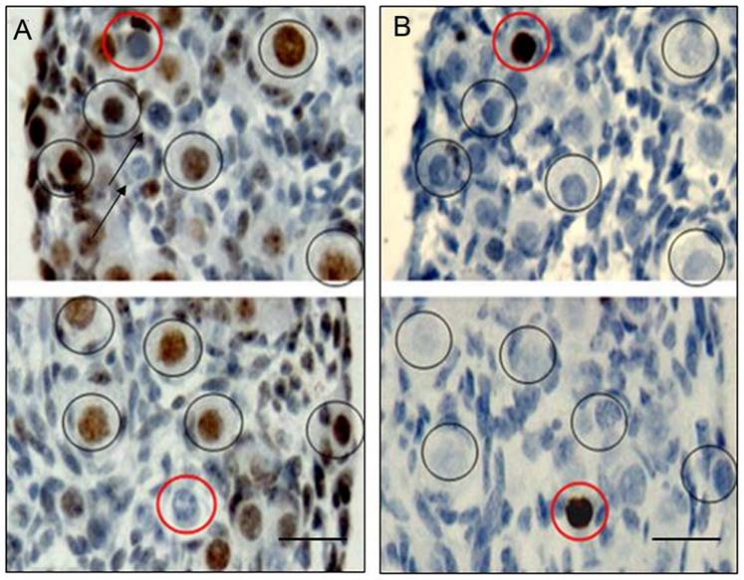
The relationship between PAR6 expression and apoptosis of germ cells. Two adjacent serial sections of 2 dpp mouse ovaries were stained by anti-PAR6 antibody (A) and TUNEL (B), respectively. PAR6 positive germ cells with negative TUNEL (black ring), while TUNEL positive germ cells with negative PAR6 (red ring). Bar = 20 µm.

In order to investigate the relationship between PAR6 expression and apoptosis of germ cells, we performed immunohistochemistry with the anti-PAR6 antibody or TUNEL at the two adjacent serial sections of new born (1 dpp-3 dpp) mouse ovaries. The nuclei of oocytes within primordial follicles and some germ cells showed positive immunolabelling for PAR6 but negative immunolabelling for TUNEL, and none of the TUNEL positive germ cells were stained with the PAR6 at any time studied (Fig. 2 and data not shown). These results indicate that the germ cells with PAR6 do not show apoptosis, and others without PAR6 may fail to be enclosed into follicles and finally undergo degeneration [21]. Some germ cells with PAR6 negative staining also showed negative immunolabelling for TUNEL (Fig. 2, arrow). The possible reason is that PAR6 reactivity loses in the germ cells prior to the time when they became positive in the TUNEL assay.

### The Anti-PAR6 antibody inhibited primordial follicle formation

**Figure 3:**
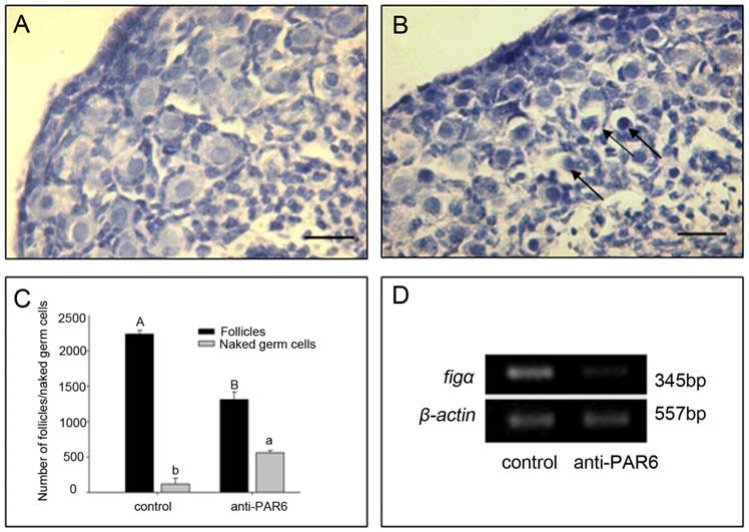
The effect of anti-PAR6 antibody on the follicle formation. 15.5 dpc ovaries were treated with anti-PAR6 antibody or goat IgG (control ) for 7 days, and then the shape (A, B), the numbers (C) of follicles and naked germ cells, and the mRNA expression of the germ cell-specific transcript factor Figα (D) were examined. The shape and number of follicles in anti-PAR6 antibody treatment were abnormal compared with that of control (arrows noted the defective follicles). Bar = 20 µm. Each follicular data came from five ovaries, and RT-PCR was repeated at least three times with similar results. Different letters were considered significant (P<0.05) respectively.

Next, experiments were carried out to further examine the role of PAR6 in the process of primordial follicle formation. According to the character of PAR6 expression, the 15.5 dpc ovaries (before PAR6 expression in germ cells) were cultured in 1 ml DMEM-F12 with 0.4 µg/ml anti-PAR6 antibody (the concentration was chosen from the histology observation, data not shown) or with 0.4 µg/ml immunoglobulin IgG from goat serum (as a negative homotype contrast) for 7 days. At the end of the culture, the numbers of follicles and naked germ cells were counted based upon traditional methods of counting in paraffin sections of the ovary [26], and the expression of the germ cell-specific transcript factor Figα (Factor in the germline, alpha) was also examined. The number of follicles declined in anti-PAR6 antibody treatment (control, 2242±29; anti-PAR6 antibody, 1312±62, P<0.05. Fig. 3) and the structures of some follicles were abnormal (Fig. 3B, arrow). However, the number of naked germ cells increased (control, 118±49; anti-PAR6 antibody, 560±19, P<0.05, Fig. 3C). Consequently, based on the counting results, the total number of germ cells declined, though not obviously. In order to determine whether more germ cells were dead, we performed TUNEL examination after culture. The result was consistent with the germ cell numbers (control, 10.59±0.36%; anti-Par6 antibody, 7.32±0.49%, P<0.05). So, in the anti-PAR6 antibody group, the apoptosis was slightly increased.

Similarly, the expression of Figα gene in the anti-PAR6 antibody treatment obviously decreased, compared with the control (Fig. 3D). Figα gene is expressed throughout the early developmental stages, including oogonia proliferation via mitosis, transformation into oocytes via meiosis, and primordial follicle formation, whereas progressive loss of its expression is closely associated with germ cell degeneration and follicle formation failure [27], [28].

### PAR6 deficiency inhibited primordial follicle formation

**Figure 4:**
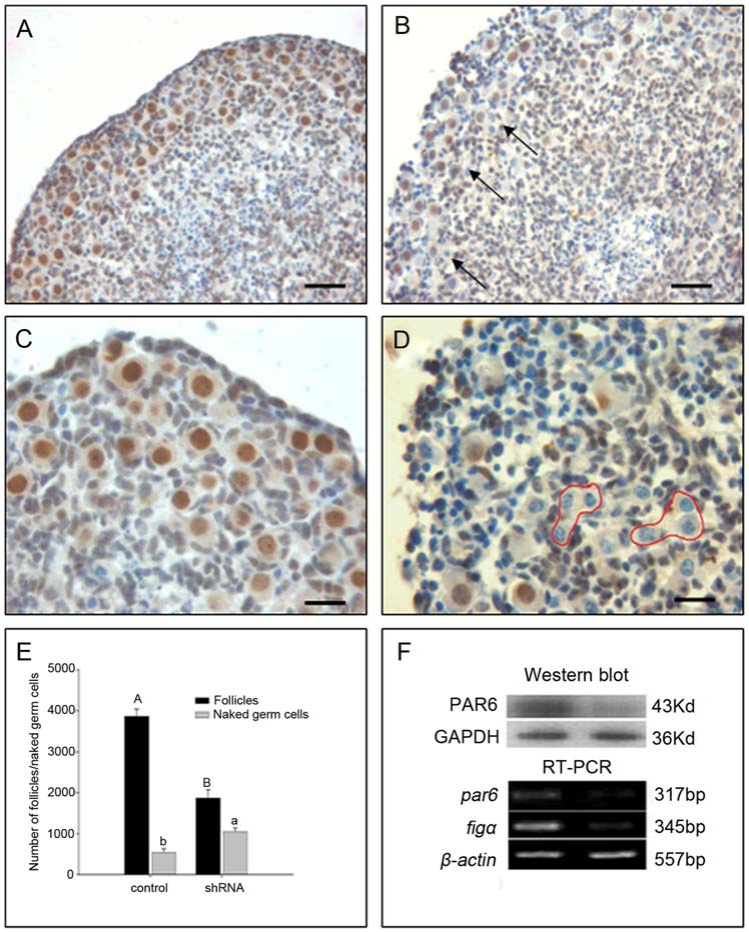
Loss of PAR6 disrupted ovarian histogenesis and reduced follicular formation. 15.5 dpc ovaries were treated with control shRNA plasmid or shRNA for 12 hours, cultured for 6.5 days, then the shape of ovaries and the localization of PAR6 (A and C, control; B and D, shRNA ),the number of follicles and naked germ cells (E), the expression of mRNA and protein (F) were examined. Arrows noted the naked germ cells and red rounds noted the cysts with PAR6 negative germ cells. Each data came from five ovaries, different superscripts denote differences (P<0.05) respectively. RT-PCR and Western blot were repeated at least three times with similar results. A and B, Bar = 40 µm; C and D, Bar = 20 µm.

For more persuasion, we employed a short hairpin RNA-mediated gene knock-down approach. We got the optimal transfection efficiency after experiments with different transfection conditions. Compared with control (Fig. 4A, C), the ovaries treated with shRNA exhibited structural disorganization (Fig. 4B,D) characterized by nests of germ cells without PAR6 (Fig. 4D, red rounds). The number of follicles declined in PAR6-deficient ovaries (control, 3865±170; shRNA, 1875±190, P<0.05, Fig. 4E), but the number of naked germ cells increased (control, 545±80; shRNA, 1055±90, P<0.05, Fig. 4E). Unfortunately, the total number of germ cells also declined like the experiment of anti-PAR6 antibody culturing. In the period of forming primordial follicles, the germ cells which couldn't be surrounded by pregranular cells would undergo apoptosis [21]. Therefore, the possibility of the reduction in total germ cell number in the anti-PAR6 antibody and shRNA groups was that the PAR6 negative germ cells cannot form primordial follicles and then died. As the RT-PCR and Western blot results showed (Fig. 4F), reducing PAR6 mRNA levels via plasmid-mediated delivery of shRNAs induced obvious decrease of Figα gene level and PAR6 protein expression. These results indicate that PAR6 is important for the formation and maintenance of primordial follicles. Although it is reported that the germline stem cells exist in mature mouse ovaries [29], there is a widely held view that the mammalian neonatal ovary contains a finite stockpile of non-growing primordial follicles, and the supply of follicles declines until advancing age, when the primordial follicular pool is exhausted [30], [31]. It needs further elucidation whether the number of the primordial follicles could be extended by enhancing the function of PAR6.

### CBX inhibited PAR6 expression in germ cells and primordial follicle formation

**Figure 5:**
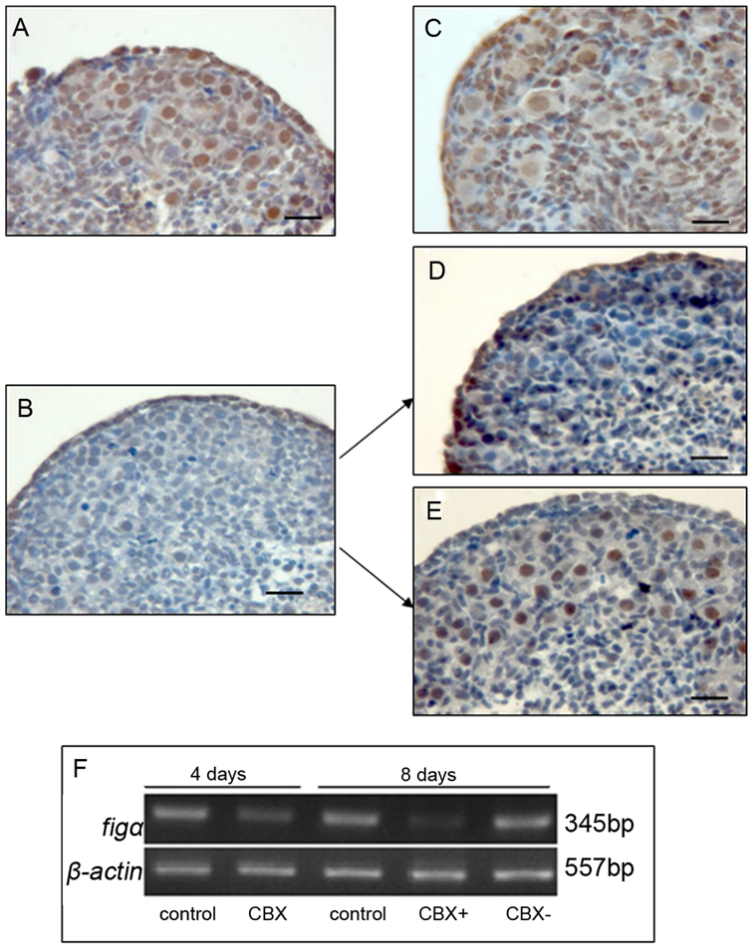
The effect of CBX on the PAR6 expression and follicles formation. 15.5 dpc ovaries were treated with or without CBX for 4 or 8 days, and then the follicular formation (A, B control; C, D, E CBX treatment) and the expression of Figα gene (F) were examined. A large number of primordial follicles were observed with strong staining of PAR6 after ovaries were cultured for 4 days (A) or 8 days (B). The germ cells stayed in cyst stage with slightly staining of PAR6 in CBX treated ovaries for 4 days (C, CBX), and no follicle was observed for 8 days (D, CBX+). Some primordial follicles were observed with strong staining of PAR6 when the ovaries were treated with CBX for 4 days and then without CBX for further 4 days (E, CBX−). Bar = 20 µm. CBX obviously decreased the mRNA level of Figα (F). The experiment was repeated at least three times with similar results. CBX, carbenoxolone.

It is worth noticing that the expression of PAR6 first occurred in the somatic cells, and decreased after 17.5 dpc when the germ cells began to express PAR6 as the somatic cells invaded cysts. A signal from the somatic cells might induce the expression of PAR6 in oocytes via gap junction communication (GJC), since the expression of connexin43 (Cx43) protein between somatic cells and germ cells is necessary for the fetal ovary development [32]. In order to examine our hypothesis, the fetal ovaries at 15.5 dpc were cultured with and without 20 µm/ml carbenoxolone (CBX, a known blocker of gap junctions) [33], [34], [35] for 4 or 8 days. The concentration was chosen from the histology observation (data not shown). The expressions of PAR6 protein and Figα gene, and the number of primordial follicles were examined at the end of the culture. A large number of primordial follicles were formed with strong expression of PAR6 in the oocyte nuclei when the ovaries were cultured for 4 days in the control group (Fig. 5A), and some growing follicles surrounded by cubical-type granulosa cells were observed with weak expression of PAR6 in the oocyte nuclei when the ovaries were cultured for 8 days (Fig. 5B). On the contrary, almost all germ cells stayed in the cyst stage after treatment with CBX for 4 days, and some of them were slightly stained for PAR6 (Fig. 5C). This inhibitory effect was further enhanced after treatment with CBX for 8 days: the germ cells still stayed in the cyst stage without obvious PAR6 expression and primordial follicle formation (Fig. 5D). Interestingly, a lot of primordial follicles were observed with strong PAR6 staining in the nuclei of oocytes when the ovaries treated with CBX for 4 days were transferred to a new culture medium without CBX for 4 more days (Fig. 5E). These results indicate that the effect of CBX can be rescued, and re-expression of PAR6 in the oocyte needs the establishment of gap junctions. Similarly, the expression of Figα gene obviously decreased after treatment with CBX for 4 days, and further decreased for 8 days treatment, but was completely recovered when the ovaries, which had been treated with CBX for 4 days, were cultured further without CBX for 4 more days (Fig. 5F).

The PAR6 (43 KDa) in germ cells might not be transferred from somatic cells via gap junctions which could only allow inorganic ions, second messengers, and small metabolites (less than about 1 KDa) to pass from cell to cell [36], [37]. In our study, CBX treatment inhibited the expression of PAR6 in germ cells possibly by blocking the signals from somatic cells to germ cells, since in Drosophila the signals from somatic cells to germ cells are required for the expression of PAR [18]. CBX also blocked the formation of primordial follicles possibly by inhibiting the expression of PAR6 for that eliminating the function of PAR6 by anti-PAR6 antibody and RNAi produced similar phenotypes of reducing the number of primordial follicles. In Drosophila, signals from the germ cells control the migration and differentiation of the somatic cells [18]. It needs further investigation whether the germ cells with the expression of PAR6 give the signals to the somatic cells through the gap junction and induce them to wrap up the germ cells to form primordial follicles in mice.

It is interesting that the expression of PAR6 in somatic cells was strong at the early stage, but weak when the somatic cells began to incorporate the cysts at 17.5 dpc. On the contrary, the nuclei of primordial follicular oocytes strongly expressed the PAR6 permanently. GCNA (germ cell nuclear antigen) is widely used as a marker for germ cells of fetal and neonatal mouse ovaries, but it is not detected in oocytes which are arrested at the diplotene stage of meiosis I [38], [39]. The PAR6 is an alternative marker for somatic cells before 17.5 dpc and for primordial follicles from the cradle to the grave.

In conclusion, the expression of PAR6 was observed in cysts when somatic cells began to invade at 17.5 dpc, and became strong in the nuclei of all primordial follicular oocytes at 3 dpp. On the other hand, it obviously decreased when the primordial follicles were recruited into the population of growing follicles. Inhibition of PAR6 by anti-PAR6 antibody, RNA interference, and CBX blocked the formation of primordial follicles. CBX also inhibited the expression of PAR6. These results indicate that the expression of PAR6 in female mouse germ cells may have new roles in contributing to the formation of primordial follicles and the maintenance of oocyte arrest at the diplotene stage. Maybe it could serve as a marker for the germ cells to be selected to form the primordial follicles. A thorough investigation of PAR6 will be useful to reveal the mechanism of primordial follicle formation and maintenance.

## Materials and Methods

### Animal (Ethics Statement)

All experiments were conducted using Kunming white mice purchased from the Laboratory Animal Center of Institute of Genetics at Beijing. Mice were housed in the facility at China Agricultural University according to the guidelines for laboratory animals approved by Beijing Experimental Animal Management Center. Adult female and male Kunming white mice were mated at late afternoon with the rate of 1∶1 to induce pregnancy and checked for vaginal plug next morning. Female breeders showing a vaginal plug on the day subsequent to mating were considered to be at day 0.5 of pregnancy.

### Fetal ovaries culture and Chemicals

Dissection of fetal ovaries was performed as described previously [40]. In brief, the pregnant mouse was killed by decapitation, fetuses were dissected out, and the urogenital region of the fetus was exposed. The ovaries were separated by microdissection from the mesonephros, decapsulated with fine needles according to their morphological difference in prechilled PBS (10 mM, PH, 7.4) under the stereomicroscope. Fetal ovaries were cultured in 24-well culture dishes (Nunclon, Nunc, Roskilde, Denmark) with 1 ml basic medium DMEM/F-12 (GIBCO, Gaithersburg, MD, USA) at 37°C, 5% CO_2_, 95% air atmosphere and saturated humidity. The developmental status was examined and recorded every 24 hours under an inverted microscopy. Anti-PAR6 antibody (Santa Cruz, CA, USA), shRNA (Santa Cruz, CA, USA), IgG (Santa Cruz, CA, USA) and CBX (Sigma, St. Louis, MO, USA) were added to the media before use.

### Immunohistochemistry

Fetal ovaries isolated at various days of pregnancy or cultured for different treatments were collected. Briefly, the tissue was fixed, embedded, and 5 mm sections were cut by a microtome. After dewaxing, rehydration and antigen retrieval with 0.01% sodium citrate buffer (pH 6.0), the sections were immunostained with primary PAR6 and MVH antibody (diluted in 1∶200, Santa Cruz) over night at 4°C. Subsequently, biotinylated secondary antibody (Zhongshan Company) and avidinbiotin-peroxidase (Zhongshan Company) were incubated, followed by exposure to diaminobenzidine (Zhongshan Company) for 1 min. Finally, the sections were counterstained with hematoxylin. Nonimmunized goat serum was used as the control.

### Ovarian follicle counts

The ovarian folliclular number was counted based upon the most widely used approach which entails that an ovary was fixed, paraffin-embedded and serially sectioned at 5 µm widths. The serial sections were placed in order on microscope slides and stained with haematoxylin. Every fifth section was analyzed for the presence of follicles, and only those follicles in which the nucleus of the oocyte was clearly visible were scored. The starting section was usually selected randomly. Finally, the cumulative follicle counts were multiplied by a factor of 5 for that four-fifths of the ovary was not analyzed.

### TUNEL test

To evaluate the relationship between PAR6 and the germ cell apoptosis, the paraffin sections of the ovaries were prepared according to the protocol described in Immunochemistry. Apoptosis was analyzed by TUNEL with In Situ Cell Death Detection Kit (Roche, Mannheim, Germany) according to the manufacturer's protocol. To determine the percentage of TUNEL positive germ cells in Anti-PAR6 antibody experiment, the number of germ cells positive for TUNEL were counted and as a percentage of the total number of germ cells in the section.

### Statistical analysis

Experiments were performed at least three times and the values are given as means±s. e. m. Data were analysed by ANOVA, using StatView software (SAS Institute, Inc., Cary, NC, USA). When a significant F ratio was defined by ANOVA, groups were compared using Fisher's protected least significant difference post hoc test. P<0.05 was considered statistically significant.

### sh RNA transfection

The shRNA and control shRNA plasmid were purchased from santa cruz. The shRNA plasmids were designed using an experimentally validated algorithm. These constructs specifically knocked down the expression of PAR6 genes by RNA interference. Each vector expressed a short hairpin RNA. Control shRNA Plasmid was a negative control for experiments using targeted shRNA transfection which encoded a scrambled shRNA sequence that would not lead to the specific degradation of any known cellular mRNA. The 15.5 dpc ovaries were transfected for 12 hours and cultured for 6.5 more days. We used control and PAR6 shRNA in accordance with the protocol. The shRNA Plasmid DNA: shRNA Plasmid Transfection Reagent ratios were begun with 1∶1 to 1∶6. The duration of transfection was 7 to 12 hours. By immunohistochemistry and RT-PCR as indicators of transfection efficiency, we found out the transfection ratio of 1 µg: 1 µl and the transfection time of 12 hours to get the optimal efficiency. Moreover, the transfection efficiency has been tested in a variety of cell lines, and there was a high efficiency as the company described. The ovaries were then fixed, serially sectioned and stained. RNA was extracted for RT- PCR and protein for Western blot.

### Western blot

In brief, total proteins from 15 ovaries were extracted in MEM-R according to manufacturer's protocol (Pierce, Rockford, IL), and the concentrations were measured by BCA procedure (CellChip. BJ Biotechnology Co., Ltd, Beijing, China). Each sample was heated to 100°C for 10 minutes. After cooling down on ice for 20 minutes and centrifuging at 12,000 rpm for 5 min, protein complexes of each sample were separated on 10% SDS–PAGE and transferred onto a piece of Protran nitrocellulose membrane (Schleicher & Schuell, Dassel, Germany). After blocking for 1 hour with 5% nonfat dry milk in TBST (20 mM Tris–HCl, 150 mM NaCl, and 0.1% Tween 20, pH 7.6), the membrane was incubated overnight at 4°C with 1∶500 goat anti-PAR6 (43 kDa) antibody. After three washes of 5 minutes each in TBST, the membrane was incubated for 1 hour at room temperature with horseradish peroxidase-conjugated rabbit anti-goat diluted 1∶5000 in TBST. After three washes in TBST, Proteins on the membrane were visualized using the enhanced chemiluminescence detection system (Amersham, Arlington Heights, IL). The levels of GAPDH were detected at the same time.

### RT-PCR

Total RNA of mouse fetal ovaries was extracted with TRIZOL Reagent (GIBCO, Gaithersburg, MD, USA). Reverse transcription (RT) was conducted with oligo (dT) using Moloney Murine Leukemia Virus Reverse Transcriptase according to the manufacturer's instruction (Promega, Madison, WI, USA). PCR amplification was performed with primers specific for each gene [28]: 5′-CTACTCCACCACGGATGACC-3′ and 5′-TTCTTCAAGCCACTCGCACA-3′ primers for Figα. Product size is 345 bp.

5′-TCAGAAACGGGCAGAAGGTG-3′ and 5′-CCAGGCGGGAGATGAAGATA-3′ primers for PAR6. Product size is 317 bp.

5′-TCCAGCCTTCCTTCTTGGGTAT-3′ and 5′-CGCCTTCACCGTTCCAGTTT-3′ primers for β-actin. Product size is 557 bp.

A total of 30 cycles were used to amplify each gene. This included a 30 seconds denaturation step at 95°C, a 30 seconds annealing step at 55°C (Figα) or 57°C (PAR6 and β-actin), and a 40 seconds extension step at 72°C. RT-PCR analysis of marker gene expression was conducted in at least three batches of samples from separated ovarian cultures with similar results presented.

## Supporting Information

Figure S1 Immunohistochemical localization of PAR6 and MVH in the adjacent section. A and B are two adjacent sections by immunohistochemistry of PAR6 and MVH (germ cell marker) at 19.5 dpc in high power field. The arrows noted the negative germ cells. Nearly all the germ cells are labeled with MVH (C) but partly with PAR6 (D) in low power field. A and B, Bar = 10 µm; C and D, Bar = 60 µm.
(0.29 MB DOC)

Figure S2 Immunohistochemical localization of PAR6 in the mouse testicle. The germ cells of the fetal and mature male did not express the PAR6. (A) 13.5 dpc; (B) 15.5 dpc; (C) 17.5 dpc; (D) 1 dpp; (E) 3 dpp; (F) 6 months. Bar = 40 µm, respectively.
(0.15 MB DOC)

## References

[1] Pepling ME (2006). From primordial germ cell to primordial follicle: Mammalian female germ cell development.. Genesis.

[2] Macara IG (2004). Par Proteins: Partners in Polarization.. Curr Biol.

[3] Telfer W (1975). Development and physiology of the oocyte-nurse cell syncitium.. Adv Insect Physiol.

[4] Deng W, Lin H (2001). Asymmetric germ cell division and oocyte determination during Drosophila oogenesis.. Int Rev Cytol.

[5] Cox DN, Seyfried SA, Jan LY, Jan YN (2001). Bazooka and atypical protein kinase C are required to regulate oocyte differentiation in the *Drosophila* ovary.. Proc Natl Acad Sci USA.

[6] Benton R, Palacios IM, St Johnston D (2002). *Drosophila* 14–3-3/PAR-5 is an essential mediator of PAR-1 function in axis formation.. Dev Cell.

[7] Lyczak R, Gomes JE, Bowerman B (2002). Heads or tails: cell polarity and axis formation in the early Caenorhabditis elegans embryo.. Dev Cell.

[8] Vaccari T, Ephrussi A (2002). The fusome and microtubules enrich Par-1 in the oocyte, where it effects polarization in conjunction with Par-3, BicD, Egl, and dynein.. Curr Biol.

[9] Martin SG, St Johnston D (2003). A role for *Drosophila* LKB1 in anterior–posterior axis formation and epithelial polarity.. Nature.

[10] Hurov JB, Watkins JL, Piwnica-Worms H (2004). Atypical PKC phosphorylates PAR-1 kinases to regulate localization and activity.. Curr Biol.

[11] Hong A, Lee-Kong S, Iida T, Sugimura I, Lilly MA (2003). The p27(cip/kip) ortholog dacapo maintains the *Drosophila* oocyte in prophase of meiosis I.. Development.

[12] Vinot S, Le T, Maro B, Louvet-Vallée S (2004). Two PAR6 proteins become asymmetrically localized during establishment of polarity in mouse oocytes.. Curr Biol.

[13] Duncan FE, Moss SB, Schultz RM, Williams CJ (2005). PAR-3 defines a central subdomain of the cortical actin cap in mouse eggs.. Dev Biol.

[14] Vinot S, Le T, Ohno S, Pawson T, Maro B (2005). Asymmetric distribution of PAR proteins in the mouse embryo begins at the 8-cell stage during compaction.. Dev Biol.

[15] Pepling ME, Spradling AC (1998). Female mouse germ cells form synchronously dividing cysts.. Development.

[16] Mackay S, Smith RA (1989). Mouse gonadal differentiation in vitro in the presence of fetal calf serum.. Cell Differ Dev.

[17] Pepling ME, Spradling AC (2001). Mouse ovarian germ cell cysts undergo programmed breakdown to form primordial follicles.. Dev Biol.

[18] Huynh JR, St Johnston D (2004). The Origin of Asymmetry: Early Polarization of the Drosophila Germline Cyst and Oocyte.. Curr Biol.

[19] Rajah R, Glaser EM, Hirshfield AN (1992). The changing architecture of the neonatal rat ovary during histogenesis.. Dev Dyn.

[20] McClellan KA, Gosden R, Taketo T (2003). Continuous loss of oocytes throughout meiotic prophase in the normal mouse ovary.. Dev Biol.

[21] Byskov AG (1986). Differentiation of mammalian embryonic gonad.. Physiol Rev.

[22] Huynh JR, Shulman JM, Benton R, St Johnston D (2001). PAR-1 is required for the maintenance of oocyte fate in Drosophila.. Development.

[23] Pellettieri J, Seydoux G (2002). Anterior-posterior polarity in C. elegans and Drosophila-PARallels and differences.. Science.

[24] Etienne-Manneville S, Hall A (2003). Cell polarity: Par6,aPKC and cytoskeletal crosstalk.. Curr Opin Cell Biol.

[25] Cheutin T, McNairn AJ, Jenuwein T, Gilbert DM, Singh PB (2003). Maintenance of Stable Heterochromatin Domains by Dynamic HP1 Binding.. Science.

[26] Tilly JL (2003). Ovarian follicle counts-not as simple as 1, 2, 3.. Repro Biol Endocrinol.

[27] Soyal SM, Amleh A, Dean J (2000). FIGa, a germ cell-specific transcription factor required for ovarian follicle formation.. Development.

[28] Lei L, Zhang H, Jin S, Wang F, Fu M (2006). Stage-Specific Germ-Somatic Cell Interaction Directs the Primordial Folliculogenesis in Mouse Fetal Ovaries.. J Cell Physiol.

[29] Johnson J, Canning J, Kaneko T, Pru JK, Tilly JL (2004). Germline stem cells and follicular renewal in the postnatal mammalian ovary.. Nature.

[30] Albertini DF (2004). Micromanagement of the ovarian reserve–do stem cells play into the ledger?. Reproduction.

[31] Greenfeld C, Flaws JA (2004). Renewed debate over postnatal oogenesis in the mammalian ovary.. Bioessays.

[32] Pérez-Armendariz EM, Sáez JC, Bravo-Moreno JF, López-Olmos V, Enders GC (2003). Connexin 43 is expressed in mouse fetal ovary.. Anat Rec A Discov Mol Cell Evol Biol.

[33] Davidson JS, Baumgarten IM, Harley EH (1986). Reversible inhibition of intercellular junctional communication by glycyrrhetinic acid.. Biochem Biophys Res Commun.

[34] Davidson JS, Baumgarten IM (1988). Glycyrrhetinic acid derivatives: a novel class of inhibitors of gap-junctional intercellular communication. Structureactivity relationships.. J Pharmacol Exp Ther.

[35] Goldberg GS, Moreno AP, Bechberger JF, Hearn SS, Shivers RR (1996). Evidence that disruption of connexon particle arrangements in gap junction plaques is associated with inhibition of gap junctional communication by a glycyrrhetinic acid derivative.. Exp Cell Res.

[36] Bruzzone R, White TW, Goodenough DA (1996). The cellular internet: On-line with connexins.. Bioessays.

[37] Bruzzone R, White TW, Paul DL (1996). Connections with connexins: The molecular basis of direct intercellular signalling.. Eur J Biochem.

[38] Enders GC, May JJ (1994). Developmentally regulated expression of a mouse germ cell nuclear antigen examined from embryonic day 11 to adult in male and female mice.. Dev Biol.

[39] Wang D, Ikeda Y, Parker KL, Enders GC (1997). Germ cell nuclear antigen (GCNA1) expression does not require a gonadal environment or steroidogenic factor 1: examination of GCNA1 in ectopic germ cells and in Ftz -F1 null mice.. Mol Reprod Dev.

[40] Wang H, Xia G, Wang Q, Li M, Lu Z (2001). Follicles were reconstituted from dissociated mouse fetal ovarian cells in vitro.. Chinese Sci Bull.

