# Supplementary figures and images for: PAR6, A Potential Marker for the Germ Cells Selected to Form Primordial Follicles in Mouse Ovary
Source: PLoS One. 2009 Oct 7;4(10):e7372. doi: 10.1371/journal.pone.0007372 (PMC2753645; doi:10.1371/journal.pone.0007372)

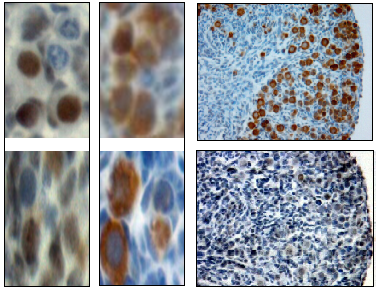


A

B

C

D

**Figure S1 Immunohistochemical localization of PAR6 and MVH in the adjacent section**

Supplement: Figure S1 — Immunohistochemical localization of PAR6 and MVH in the adjacent section. A and B are two adjacent sections by immunohistochemistry of PAR6 and MVH (germ cell marker) at 19.5 dpc in high power field. The arrows noted the negative germ cells. Nearly all the germ cells are labeled with MVH (C) but partly with PAR6 (D) in low power field. A and B, Bar = 10 µm; C and D, Bar = 60 µm. (0.29 MB DOC) [file pone.0007372.s001.doc]

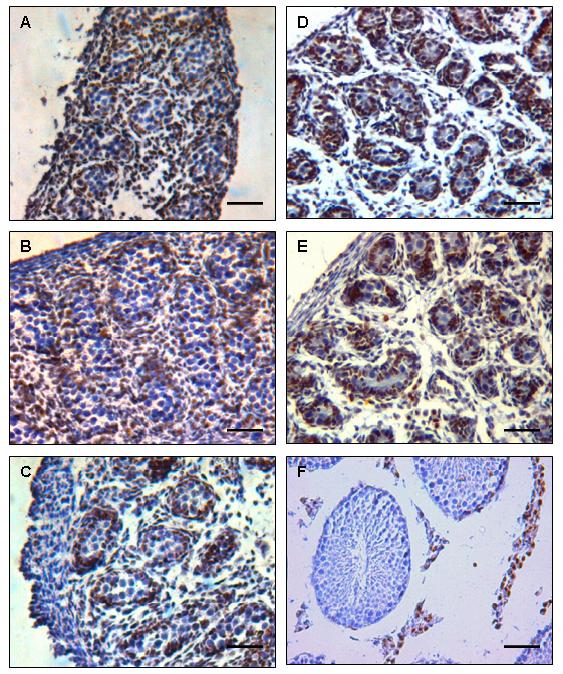


**Figure S2 Immunohistochemical localization of PAR6 in the mouse testicle.**

Supplement: Figure S2 — Immunohistochemical localization of PAR6 in the mouse testicle. The germ cells of the fetal and mature male did not express the PAR6. (A) 13.5 dpc; (B) 15.5 dpc; (C) 17.5 dpc; (D) 1 dpp; (E) 3 dpp; (F) 6 months. Bar = 40 µm, respectively. (0.15 MB DOC) [file pone.0007372.s002.doc]
